# The Cognitive Effects of Radiotherapy for Brain Metastases

**DOI:** 10.3389/fonc.2022.893264

**Published:** 2022-06-30

**Authors:** Eric J. Lehrer, Brianna M. Jones, Daniel R. Dickstein, Sheryl Green, Isabelle M. Germano, Joshua D. Palmer, Nadia Laack, Paul D. Brown, Vinai Gondi, Jeffrey S. Wefel, Jason P. Sheehan, Daniel M. Trifiletti

**Affiliations:** ^1^ Department of Radiation Oncology, Icahn School of Medicine at Mount Sinai, New York, NY, United States; ^2^ Department of Neurosurgery, Icahn School of Medicine at Mount Sinai, New York, NY, United States; ^3^ Department of Radiation Oncology, Ohio State University Wexner Medical Center, Columbus, OH, United States; ^4^ Department of Radiation Oncology, Mayo Clinic, Rochester, MN, United States; ^5^ Department of Radiation Oncology, Northwestern Medicine Cancer Center Warrenville and Proton Center, Warrenville, IL, United States; ^6^ Department of Radiation Oncology, MD Anderson Cancer Center, Houston, TX, United States; ^7^ Department of Neurological Surgery, University of Virginia, Charlottesville, VA, United States; ^8^ Department of Radiation Oncology, Mayo Clinic, Jacksonville, FL, United States

**Keywords:** brain metastases, cognition, radiation therapy, radiosurgery, whole brain radiation therapy, neurosurgery, neuro-oncology, radiation oncology

## Abstract

Brain metastases are the most common intracranial neoplasm and are seen in upwards of 10-30% of patients with cancer. For decades, whole brain radiation therapy (WBRT) was the mainstay of treatment in these patients. While WBRT is associated with excellent rates of intracranial tumor control, studies have demonstrated a lack of survival benefit, and WBRT is associated with higher rates of cognitive deterioration and detrimental effects on quality of life. In recent years, strategies to mitigate this risk, such as the incorporation of memantine and hippocampal avoidance have been employed with improved results. Furthermore, stereotactic radiosurgery (SRS) has emerged as an appealing treatment option over the last decade in the management of brain metastases and is associated with superior cognitive preservation and quality of life when compared to WBRT. This review article evaluates the pathogenesis and impact of cranial irradiation on cognition in patients with brain metastases, as well as current and future risk mitigation techniques.

## Introduction

Current estimates indicate that roughly 200,000 patients are diagnosed with brain metastases annually in the United States, and 10-30% of patients with cancer receive a diagnosis of brain metastases during their disease course (1–4). These estimates may be conservative, as the true incidence is likely higher, due to a multitude of factors, such as undiagnosed brain metastasis identified on autopsy and underreporting with national registries (e.g. The National Cancer Database and Surveillance, Epidemiology, and End Results) (5, 6). Historically, patient prognosis was poor with a median overall survival of 3-4 months in patients who did not undergo surgical intervention (7). However, advancements in systemic therapy, surgery, and radiation therapy have resulted in survival advantages across multiple malignancies, thus less common and aggressive histologies are increasingly metastasizing to the brain (e.g., gastrointestinal primary cancers) (1, 8–13). Additionally, the widespread availability of MRI imaging has enhanced detection of subclinical disease.

Whole-brain radiation therapy (WBRT) is a treatment modality that has been used since the 1950s for patients with brain metastases (14). It is commonly delivered to a total dose of 30 Gy over 10 sessions. Clinicians traditionally favored WBRT due to its efficacy in providing palliation and ability to target unknown microscopic intracranial disease. WBRT has been shown to result in improved intracranial tumor control in multiple randomized trials; however, WBRT has also been shown to result in significant cognitive decline, which has been observed in up to 50% of patients following treatment (15–20). These patients can present with one or multiple cognitive domains affected, such as executive function, learning and memory, processing speed, and verbal fluency. As the prognosis in patients with brain metastases continues to improve, treatment has increasingly focused on preservation of quality of life (QOL) and cognitive function. Multiple studies have suggested that there is a correlation between neurocognitive functioning and QOL (21, 22). In recent years, the addition of memantine and hippocampal avoidance to WBRT have demonstrated significant preservation of cognitive sequelae and, in the setting of hippocampal avoidance, better preservation of patient-reported QOL, and now represent current standard of care in appropriately selected patients (17, 23, 24).

Moreover, the efficacy of brain-directed radiotherapy in providing adequate palliation has come under question in recent years. In 2016, the QUARTZ trial, which randomized 538 patients with non-small cell lung cancer brain metastases and poor prognosis, to dexamethasone with WBRT or dexamethasone with supportive care alone was published (25). This trial concluded that WBRT did not offer any benefit in QOL or survival (median survival in both arms was approximately 2 months) over supportive care, thus calling into question the efficacy of brain-directed radiotherapy in this setting. However, while broad, indiscriminate use of WBRT has fallen out of favor, it continues to be a commonly used modality in patients with a high intracranial burden of brain metastases (26, 27).

Another important advancement in the treatment of brain metastases is stereotactic radiosurgery (SRS), which is defined as the delivery of a high dose of very conformal radiation in 1-5 fractions with marked sparing of nearby healthy tissues (28). SRS has been shown to result in fewer cognitive side effects than conventional WBRT (15, 16, 18). However, WBRT has been shown to provide superior rates of intracranial control, especially by decreasing the risk of development of new brain metastases, when compared to SRS (15, 16, 18, 29). As a result, there is some controversy regarding the use of SRS in patients with large numbers of brain metastases (30, 31). Multiple studies have reported a lower risk of cognitive decline with SRS than conventional WBRT; studies comparing SRS to contemporary WBRT with neuroprotective strategies, such as memantine and hippocampal avoidance remain ongoing (32, 33).

In this article, we review the pathogenesis, diagnosis, and evaluation of cognitive decline following cranial irradiation. Additionally, we review the role of SRS, hippocampal avoidance, and memantine as risk mitigation strategies in patients undergoing cranial irradiation for brain metastases.

## Pathogenesis of Cognitive Decline Following Radiation Therapy

Despite evidence showing that radiation causes cognitive impairment, the pathophysiological understanding of this common clinical scenario is poorly understood. The cause is believed to be multifactorial with changes in brain vasculature, stem-cell depletion, and changes to the brain’s microenvironment all being implicated. While damage to the hippocampus has been implicated in cognitive decline following cranial irradiation, recent evidence suggests that damage to white matter and other cortical territories, such as the frontal cortex also play a significant role (34–37).

### Cerebrovascular Damage

Vascular pathology has been associated with many neurodegenerative diseases. It is hypothesized that one of the contributing mechanisms to Alzheimer’s Disease is the weakening of blood vessels due to the accumulation of amyloid-beta plaques in vessel walls (38). Similarly, radiation therapy can cause damage to vascular endothelial cells (39–41). In a rodent model, 10 weeks following completion of cranial irradiation to 40 Gy (5 Gy twice weekly over 4 weeks) notable changes were observed in blood vessel length and density (42). These findings suggest that ionizing radiation has the potential to cause persistent vascular damage which is frequently observed in neurocognitive diseases.

Radiation therapy can also cause blood brain barrier disruption with resultant edema (43). This can lead to abnormalities in the brain’s microenvironment and microvasculature, which have been implicated in the pathogenesis of cognitive decline (43–45). This process is largely due to apoptosis in response to increased ceramide production (46). Studies have demonstrated that radiation doses as low as 5 Gy result in the production of ceramides (46–48). Additionally, experiments using murine models have demonstrated that ionizing radiation can lead to cerebrovascular damage within in the hippocampus, which persists following completion of treatment (49, 50). These findings suggest that ionizing radiation can lead to permanent dysfunction of angiogenesis in the hippocampus, which is the primary brain region responsible for learning and memory.

### Neuroanatomical Changes

Alterations in neuronal morphology and structure has been linked to both neurological and psychiatric disorders as well as to normal aging (51). The morphology of neuronal dendritic spines, which serve as the site of synaptic transmission, are believed to play a role in neuropsychiatric disorders, as well as cognition (52, 53). Dendritic spines also contain N-methyl-D-aspartate (NMDA) glutamate receptors, which allow for calcium influx into cells, and play a major role in learning and memory. Thus, dendritic spine morphologies with greater surface area contain a higher concentration of NMDA receptors, resulting in greater synaptic strength (54, 55). Multiple studies have demonstrated that dendritic spine and neuronal architecture play an integral role in normal aging, as well as multiple neurologic diseases and developmental disorders (56–60).

Several studies have demonstrated that ionizing radiation alters dendritic spine density and morphology, as well as neuronal architecture (61–63). In 2013, a study by Parihar et al. utilizing a murine model demonstrated significant reductions in dendritic spine complexity (>50%), frequency (20-35%), and density (40-70%) on hippocampal neurons of the dentate gyrus in response to cranial irradiation in a dose-dependent manner (63). In 2018, a study by Duman et al. using a murine model demonstrated that the administration of memantine prior to cranial irradiation can prevent radiation-induced synaptic remodeling (64). Taken together, these findings suggest that ionizing radiation can alter neuronal anatomy and NMDA receptor density, both of which are associated with cognitive decline. Additionally, memantine may play a protective role in this setting.

### Impairment of Neurogenesis

The anatomical components of the hippocampus include the dentate gyrus, CA3, and CA1 regions, and the subventricular zone. The subgranular zone of the dentate gyrus is the site of hippocampal neurogenesis (65, 66). This process is an integral component of cognition and memory (67, 68). In 2013, a study by Boström et al. demonstrated that the delivery of 8 Gy to the brain of young mice resulted in decreased density of neural stem and progenitor cells, while the vasculature normalized over time (69). These findings suggest that ionizing radiation leads to decreased hippocampal neurogenesis. While the exact mechanism of neural stem cell death is not fully understood, it has been hypothesized that it occurs *via* apoptosis due to JNK pathway activation (70).

### Neuroinflammation

Cranial irradiation activates astrocytes and microglia leading to neuroinflammation and reactive gliosis (71). Upregulation of pro-inflammatory chemokines, including CCL2, IL-6, IL-18, IL-1α, TNF-α; reactive oxygen species; and nitric oxide, in response to cranial irradiation play a major role in activation of these CNS cell types (72–74). Additionally, microglia, which normally aid in neuroprotection and synapse integrity, will release neurotoxic factor which induces neuronal cell death and contributes to cerebral edema (75, 76). Increased TNF-α activity has been shown to lead to blood brain barrier breakdown and immune cell activation (77). In 2012, a study by Belarbi et al. demonstrated that anti-TNF-α agents were able to restore neuronal function and reverse cognitive deficits due to chronic neuroinflammation in rats (73). A subsequent study demonstrated that inhibition of microglia mediated neuroinflammation in response to cranial irradiation in mice results in improved cognitive function (78). These findings suggest that neuroinflammation in response to cranial irradiation plays a key role in the pathogenesis of cognitive decline following treatment.

## Patient Presentation

It is important to note that patients with brain metastases typically have cognitive impairment at baseline before radiotherapy: a phase 3 trial with prospective cognitive testing found greater than 90% of patients had impairment on one of more cognitive tests at baseline (20). Following completion of radiotherapy, patients can present with cognitive decline as early as 1- to 6-months following treatment. Symptom presentation during this time window is potentially reversible and is believed to be due to transient demyelination (43). Patients who present at 6-months or later generally have irreversible cognitive dysfunction with multiple affected cognitive domains. These patients frequently present with deficits in attention, information processing, executive function, and learning and memory. Multiple radiographic findings, such as white matter abnormalities and changes in fractional anisotropy on diffusion tensor MRI have shown an association with cognitive decline (79–91). Consultation with neuropsychologists can be very helpful in quantifying and trending cognitive changes. Additionally, it is essential for clinicians to rule out other possible causes, such as dementia, delirium, metabolic and endocrinologic disturbances, and disease progression.

## Neurocognitive Assessment

The diagnosis of neurocognitive decline following cranial irradiation requires neuropsychological assessment. Earlier clinical trials utilized the screening test, the Mini-Mental Status Exam (MMSE); however, its use in this setting has fallen out of favor due to its limited sensitivity in diagnosing cognitive impairment (92). More commonly, clinical trials now employ neuropsychological testing that assesses multiple cognitive domains, such as executive function, learning and memory, verbal fluency, and attention. Commonly utilized cognitive assessments on randomized phase 3 clinical trials are presented in **Table 1**.

**Table 1:** Phase 3 Studies Utilizing Stereotactic Radiosurgery or Memantine/Hippocampal Avoidance in Patients with Brain Metastases Incorporating Neuropsychological Testing.

Study	Treatment Arms	Treatment Details	Cognitive Domains/Tests	Cognitive Outcomes
MDACC (2009) (18)	SRS + WBRT (n = 28)	SRS: Dose based on tumor diameter as per 90-05 (93) < 2 cm: 20 to 24 Gy 2-3 cm: 18 Gy 3-4 cm: 15 Gy WBRT: 30 Gy in 12 fractions	Attention: WAIS-III Digit Span Processing speed: WAIS-III Digit Symbol, TMT-A Learning and memory: HVLT-R Verbal fluency: COWA Executive function: TMT Part B Upper extremity fine motor dexterity: Lafayette Grooved Pegboard	Significant drop in HVLT-R Total Recall at 4 months (mean posterior probability of decline of 52% for SRS + WBRT vs 24% in SRS alone group), which was persistent at 6 months (28% vs 8%) Significant drop in HVLT-R Delayed Recall for SRS + WBRT vs SRS alone (22% vs 6%) and HVLT-R Delayed Recognition (11% vs 0%), respectively at 4 months Significant drop in executive function (COWA, TMT B) in the SRS + WBRT group compared to SRS alone group
	SRS (n = 30)			
RTOG 0614 (2013) (17)	WBRT + Memantine (n = 256)	WBRT: 37.5 Gy in 15 fractions Memantine: Week 1: 5 mg PO QD Week 2: 5 mg PO BID Week 3: Morning dose increased to 10mg Target dose for weeks 4 through 24: 10mg BID	Learning and Memory: HVLT-R Processing speed: TMT-A Executive function: TMT-B Verbal fluency: COWA MMSE	Less decline in HVLT-R Delayed Recall in memantine arm but not statistically significant at 8 weeks (*p* = 0.069) and at 24 weeks (*p* = 0.059) Less decline in HVLT-R Delayed Recall (raw and standardized scores; *p* = 0.0149, *p* = 0.0115), MMSE (raw scores, *p* = 0.0093) at 24 weeks in memantine arm Less decline in COWA (2 SD decline; *p* = 0.0015) at 8 weeks in memantine arm Time to cognitive failure, defined as the first cognitive failure on any of the neurocognitive tests, favored memantine arm (*p* = 0.01) Rate of cognitive decline over time slowed by 4 months after WBRT in both arms, but more so in memantine arm
	WBRT (n = 252)			
N0574 (2016) (16)	SRS + WBRT (n = 102)	SRS: 18-22 Gy WBRT: 30 Gy in 12 fractions	Learning and immediate memory: HVLT-R IR Upper extremity fine motor dexterity: Lafayette Grooved Pegboard Verbal fluency: COWA Processing speed: TMT-A Executive function: TMT-B	Less cognitive deterioration (defined as a decline of greater than 1 SD from baseline on at least 1 of the 7 cognitive tests) at 3 months after SRS alone (63.5% vs. 91.7%; *p* < 0.001) Significant decline for HVLT-R Total Recall SRS + WBRT vs SRS alone (30.4% vs 8.2%; *p* = 0.004), HVLT-R Delayed Recall (51.1% vs 19.7%; *p* < 0.001), and COWA (18.6% vs. 1.9%, *p* = 0.01), respectively
	SRS (n = 111)	SRS: 20-24 Gy		
N107C (2017) (15)	Surgery + SRS (n = 98)	SRS: 12 to 20 Gy (volume-based) < 4.2 cm^3^: 20 Gy 4.2-7.9 cm^3^: 18 Gy 8.0-14.3 cm^3^: 17 Gy 14.4-19.9 cm^3^: 15 Gy 20.0-29.9 cm^3^: 14 Gy ≥30.0 cm^3^ (up to 5 cm diameter): 12 Gy	Learning and immediate memory: HVLT-R IR Upper extremity fine motor dexterity: Lafayette Grooved Pegboard Verbal fluency: COWA Processing speed: TMT-A Executive function: TMT-B	Median cognition-deterioration-free survival longer after SRS to surgical cavity than after WBRT (3.7 vs 3.0 months; *p* < 0.0001) At 6 months, patients in the SRS arm had less overall cognitive deterioration (52% vs 85%; *p* = 0.00031)
	Surgery + WBRT (n = 96)	WBRT: 30 Gy in 10 fractions or 37.5 Gy in 15 fractions		
NRG CC001 (2020) (23)	HA-WBRT + Memantine (n = 261)	WBRT: 30 Gy in 10 fractions Memantine: Same dosing schedule as RTOG 0614	Learning and memory: HVLT-R Verbal fluency: COWA Processing speed: TMT-A Executive function: TMT-B	Time to cognitive failure (defined as cognitive decline determined by reliable change index on at least one of the cognitive tests) significantly lower in HA-WBRT + memantine arm compared with WBRT + memantine arm (*p* = 0.03) HA-WBRT + memantine arm less likely to have deterioration in TMT-B (*p* = 0.01), HVLT-R Total Recall (*p* = 0.049) and HVLT-R Delayed Recognition (*p* = 0.02) at 6 months
	WBRT + Memantine (n = 257)			

BID, twice daily; COWA, Controlled Oral Word Association; Gy, gray; HVLT-R, Hopkins Verbal Learning Test - Revised; LINAC, linear accelerator; MMSE, mini-mental state exam; PO, by mouth; QD, once daily; SRS, stereotactic radiosurgery; TMT-A, Trail Making Test Part A; TMT-B, Trail Making Test Part B; WAIS-III, Wechsler Adult Intelligence Scale-Third Edition; WBRT, whole-brain radiation therapy; HA-WBRT, hippocampal avoidance whole-brain radiation therapy.

## Treatment Strategies to Mitigate the Risk of Cognitive Decline

### Stereotactic Radiosurgery

**Figure 1:**
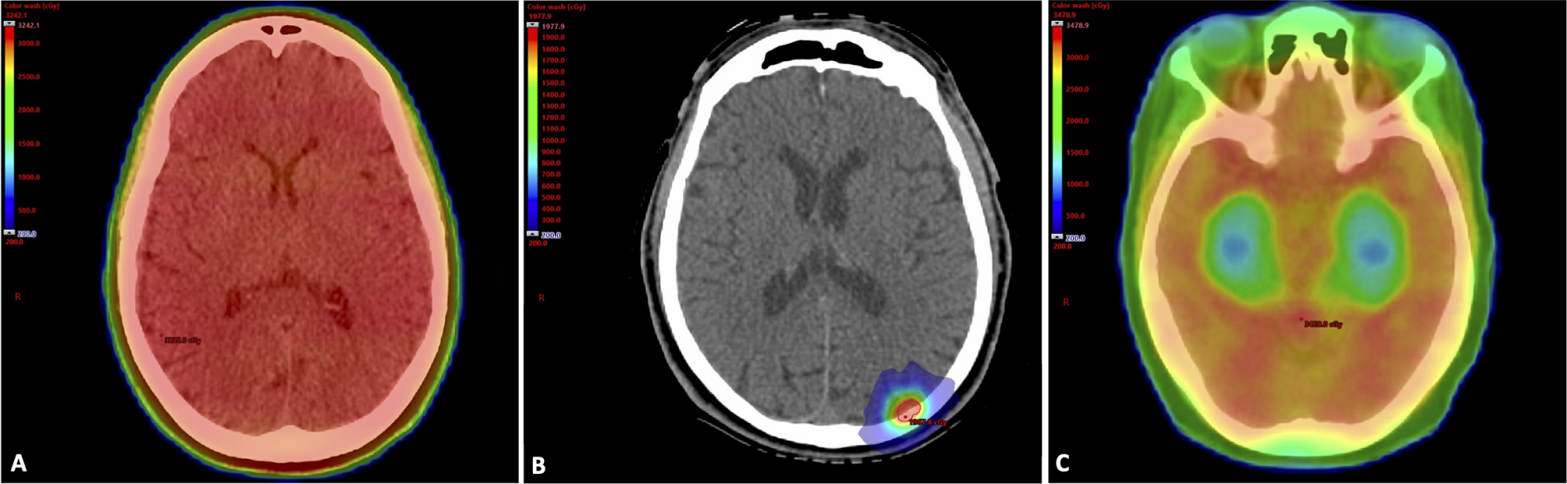
**(A)** Whole Brain Radiation Therapy Treatment Plan. Treatment plan for a 65-year-old woman with metastatic non-small cell lung cancer. She had a large burden of intracranial disease and was treated with WBRT to a dose of 30 Gy in 10 fractions. In WBRT, the entire brain including areas that play a major role in neurocognition receive the prescription radiation dose. The patient received memantine during and after treatment based on dosing from RTOG 0614. (Gy, gray; RTOG, radiation therapy oncology group; WBRT, whole brain radiation therapy). **(B)** Stereotactic Radiosurgery Treatment Plan. Treatment plan overlaid on simulation CT scan for a 50-year-old man with a history of BRAF wild-type metastatic melanoma who developed a left occipital lobe metastasis. He was treated with single fraction SRS to a dose of 20 Gy. (Gy, gray; SRS, stereotactic radiosurgery). **(C)** Whole Brain Radiation Therapy with Hippocampal Avoidance Treatment Plan. Treatment plan for a 60-year-old woman with metastatic breast cancer who was treated with HA-WBRT and memantine to 30 Gy in 10 fractions. Areas in red received the prescription dose, while areas in green and blue represent lower dose areas. Note the sparing of the bilateral hippocampi. (Gy, gray; HA-WBRT, hippocampal avoidance whole brain radiation therapy).

The delivery of conventional WBRT typically involves the use of two opposed lateral radiation fields resulting in the entire brain receiving the prescription radiation dose, as shown in **Figure 1A**. The ability to reduce dose to areas that play a central role in neurocognition is an effective strategy to mitigate the risk of cognitive decline following irradiation. Stereotactic radiosurgery allows for the treatment of an intracranial target while largely sparing healthy surrounding tissues and, for brain metastasis, has demonstrated excellent rates of local tumor control and improved neurocognition following treatment when compared to conventional WBRT in the randomized setting (15, 16, 18) **(** **Figure 1B** **)**. The details of several of these trials are presented in **Table 1**.

In 2009, Chang et al. published a randomized phase 3 trial conducted at the MD Anderson Cancer Center (18). Patients with 1-3 newly diagnosed brain metastases were randomly assigned to receive SRS alone or SRS + conventional WBRT. All patients underwent formal neuropsychological assessments. With a median follow-up 9.5 months, the trial was closed early due to a 95% probability that patients in the SRS + conventional WBRT arm were more than twice as likely to experience learning and memory deficits at 4 months versus the SRS alone arm. In 2016, Brown et al. published the results of a multicenter phase 3 trial conducted across 34 institutions in North America (16). This study randomized 213 patients with 1-3 brain metastases to receive SRS + conventional WBRT or SRS alone. With a median follow-up of 7.2 months, the rate of cognitive deterioration at 3-months was 63.5% versus 91.7% (*p* < 0.001), favoring the SRS arm. Additionally, no difference in overall survival were observed. Taken together, the findings of these trials suggest that in patients with 1-3 brain metastases, the use of SRS alone may be the preferred treatment strategy, as it minimizes cognitive decline with no detriment to patient survival.

The impact of SRS and conventional WBRT on neurocognition has also been evaluated in the adjuvant setting. In 2017, Brown et al. published the results of the N107C trial, which was a phase 3 study that randomized 194 patients across 48 North American institutions with a single resected brain metastasis to receive adjuvant SRS or conventional WBRT (15). Patients in the SRS arm experienced superior median cognitive-deterioration-free survival compared to the conventional WBRT arm (3.7 months vs. 3.0 months; *p* < 0.001). No survival difference was observed. Additionally, overall cognitive deterioration was higher in the conventional WBRT arm (52% vs. 85%; *p* = 0.00031). At 12-months, surgical bed control rates were 60.5% vs. 80.6% (*p* = 0.00068), favoring the conventional WBRT arm. This may be due to a large proportion of patients (40% in each arm) having a resection cavity diameter > 3 cm. These findings suggest that in the adjuvant setting, SRS results in improved cognitive preservation as compared to conventional WBRT. Furthermore, in the setting of larger surgical cavities or larger intact metastases, fractionated radiosurgery may be a viable alternative to maximizing local control while preserving neurocognition (94–96).

While SRS has been associated with lower rates of cognitive decline, multiple randomized controlled trials have demonstrated that SRS alone is associated with inferior local and distant brain control compared to therapeutic strategies incorporating WBRT (15, 16, 18). On the N0574 trial, time to intracranial failure was significantly shorter in the SRS compared to the SRS + conventional WBRT arm [hazard ratio (HR): 3.6; 95% confidence interval (CI): 2.2-5.9; *p* < 0.001]. Additionally, at 6 months, the local control rates were 81.6% versus 92.6%, favoring the SRS + WBRT arm (*p* = 0.034). Distant brain control rates at 6 months were 76.7% versus 94.7%, favoring the SRS + conventional WBRT arm (*p* < 0.001) (16). Similar findings were observed in the adjuvant setting on the N107C trial, where the 6-month surgical bed control was 80.4% versus 87.1%, favoring the conventional WBRT arm (*p* < 0.001) (15). Distant brain control rates at 6 months were 72.1% versus 94.6%, favoring the conventional WBRT arm (*p* < 0.001). Thus, conventional WBRT is associated with improved intracranial tumor control compared to SRS, which is likely due to irradiation of subclinical disease.

### Memantine

Memantine is an antagonist of the NMDA receptor, which is a voltage-gated glutamate receptor that allows calcium entry into cells. It is presently FDA approved for use in moderate to severe Alzheimer dementia. In 2013, Brown et al. published the results of RTOG 0614, which was a phase 3 trial that randomized 508 patients with brain metastases to receive WBRT with or without the addition of memantine (17). Memantine was administered over the course of 24 weeks with the following dosing: (1) Week 1: 5 mg PO in the morning; (2) Week 2: 5 mg PO in the morning and 5 mg PO in the evening; (3) Week 3: 10 mg PO in the morning and 5 mg PO in the evening; and (4) Weeks 4-24: 10 mg in the morning and 10 mg in the evening. Patients completed formal neuropsychological testing and MMSE at regular follow-up intervals. The primary endpoint was whether memantine preserved cognitive function at 24 weeks measured by the Hopkins Verbal Learning Test – Revised (HVLT-R) Delayed Recall. The median follow-up was 12.4 months. At 24 weeks, there was less cognitive decline in the memantine arm compared to placebo; however, this was not statistically significant (*p* = 0.059). This was likely due to there only being 149 patients analyzable at 24 weeks, lowering the statistical power to detect a difference to 35%. Time to cognitive failure, which incorporated multiple cognitive domains was statistically significant and favored the memantine arm (HR: 0.784; 95% CI: 0.621-0.988; *p* = 0.01). There were no statistically significant differences in grade 3-4 toxicities, progression-free survival, or overall survival between the study arms.

While the primary endpoint was not statistically significant, these results need to be interpreted in a modern context. First, these patients were treated between 2008-2010, this was prior to the advent of immune checkpoint inhibitors, which have markedly improved survival in multiple advanced malignancies (97–101). As a result, patients treated today would be more likely to live longer and would therefore be able to complete follow-up cognitive assessments. Second, the dominant benefit in time to cognitive failure was not apparent until approximately 3 months after completing WBRT. Therefore, patients with shorter follow-up times likely had poorer baseline prognostic factors and experienced early disease progression. This suggests that memantine is likely more beneficial in patients with a better prognosis and life expectancy. Third, the primary endpoint only accounted for cognitive decline as measured by a decrease in delayed recall on the HVLT-R Delayed Recall. Therefore, time to cognitive failure, which was a composite endpoint that accounted for multiple cognitive domains is likely more clinically meaningful and did show a significant benefit with the addition of memantine to WBRT. Taken together, the findings of RTOG 0614 suggest that memantine has the potential to reduce cognitive decline in patients undergoing WBRT without an increased risk of toxicity and is therefore considered standard of care in patients receiving WBRT.

When prescribing memantine, clinicians should discuss the potential side effects, such as headache, confusion, dizziness, nausea, and agitation. Additionally, caution should be exercised when patients have a history of liver and renal impairment.

## Hippocampal Avoidance

Due to the role the hippocampus plays in learning and memory, there has been a great deal of interest in sparing this region of the brain during WBRT (23, 24, 102). In 2014, Gondi et al. published the results of RTOG 0933, which was a phase 2 multi-institutional study where patients with brain metastases outside a 5 mm margin around the hippocampi received WBRT with hippocampal avoidance to a dose of 30 Gy in 10 fractions **(** **Figure 1C** **)** (24). There were 100 patients enrolled and all underwent formal neuropsychological testing. Enrolled patients were compared to the control arm of PCI-P-120-9801, which was a phase 3 study utilizing WBRT with identical eligibility criteria to RTOG 0933 (103). At 4 months, the mean relative decline in the modified HVLT-R Delayed Recall compared to baseline was 7%, which was significantly improved from the historical control (*p* < 0.001). In addition, cognitive results were comparable to what had been observed in prior studies of SRS (18). Similar to the findings of RTOG 0614, the benefits in cognitive preservation were seen in patients who were able to complete neuropsychological testing at 4 months. Thus, hippocampal avoidance is likely more beneficial in patients with a better baseline prognosis and life expectancy.

In 2020, Brown et al. published the results of NRG CC001, which was a phase 3 trial where patients with brain metastases were randomized to: (1) hippocampal avoidance WBRT with memantine; or (2) WBRT with memantine (23). There were 518 patients enrolled with a median follow-up of 7.9 months. All patients completed neuropsychological testing at regular intervals. The primary endpoint was time to cognitive failure, as shown in **Table 1**. The risk of cognitive failure favored the hippocampal avoidance arm (HR: 0.76; 95% CI: 0.60-0.98; *p* = 0.03). Additionally, at 6-months patients in the hippocampal avoidance arm had less memory complaints (*p* = 0.01), fewer cognitive symptoms (*p* = 0.01), and less symptom interference (*p* = 0.008). At 6-months, approximately 80% of patients in each arm died. This suggests that patients with a better baseline prognosis and life expectancy are likely to benefit the most from hippocampal avoidance. However, not all patients with brain metastases were eligible for inclusion on NRG CC001, such as patients with ventricular system distortion or hydrocephalus, the presence of leptomeningeal disease, and brain metastases arising from primary germ cell tumors, small cell carcinoma, an unknown primary or lymphoma. In 2021, a phase 2 randomized trial conducted in China compared WBRT with or without the use of hippocampal avoidance in patients with brain metastases (104). This trial demonstrated that hippocampal avoidance as associated with better memory preservation at 6-months compared to conventional WBRT.

Taken together, these trials suggest that in patients undergoing WBRT, the use of memantine and hippocampal avoidance reduces the risk of cognitive decline following WBRT. Additionally, these benefits are the most apparent in patients with a better baseline prognosis and life expectancy. However, not all patients are eligible for hippocampal avoidance, as no metastases are permitted within 5 mm of the bilateral hippocampi. Furthermore, hippocampal avoidance requires the use of advanced planning methods, such as intensity modulated radiation therapy and volumetric modulated arc therapy. Similarly, SRS requires specialized planning techniques, as well as specialized radiosurgery platforms to deliver treatment. Therefore, not all centers may have the necessary technical capabilities to deliver these treatments, particularly in underserved areas.

## Future Directions

Preserving neurocognition in patients undergoing cranial irradiation continues to be a major area of research focus. In patients with small cell lung cancer (SCLC), prophylactic cranial irradiation (PCI) is frequently administered to a dose of 25 Gy in 10 fractions in patients with no detectable brain metastases (105–107). PCI was historically administered using conventional WBRT techniques; however, recent studies have assessed incorporating hippocampal avoidance in this setting. In 2021, the PREMER study was published, which randomized 150 patients with SCLC to standard or hippocampal avoidance PCI across 13 institutions in Spain (108). At 3-months the investigators observed that the decline in memory favored the hippocampal avoidance arm (5.8% vs. 23.5%; *p* = 0.003). However, in 2021, a phase 3 trial conducted in the Netherlands did not observe a lower probability of cognitive decline in the hippocampal avoidance PCI arm (109). The NRG CC003 trial is presently investigating this hypothesis in North America and will be completing accrual later this year (110).

While there is strong evidence supporting the role of SRS in the management of limited numbers of brain metastases (15, 16, 18), the use of SRS remains controversial in the management of larger numbers of lesions. In 2020, a study by Rinna et al. observed that in patients undergoing Gamma Knife ^®^ SRS that 10 or more metastases, and metastases in close proximity to the hippocampi were at an increased risk for excessive hippocampal dosing (111). In 2021, a study published by Burgess et al. evaluating 60 SRS plans with a median distance to the hippocampus of 2.4 cm observed that patients can undergo replanning to decrease the hippocampal dose by > 50% without compromising target coverage (112). Taken together, these findings suggest that limiting dose to the hippocampus during SRS may further decrease the risk of cognitive decline in these patients.

CCTG CE.7 is an ongoing phase 3 trial that is randomizing patients with 5-15 brain metastases to SRS or WBRT with the addition of memantine and hippocampal avoidance (33). There is ongoing prospective investigation into the role that regions outside of the hippocampus play in cognitive decline following cranial irradiation (113). There is presently a trial underway at Johns Hopkins University investigating neurocognitive functioning with sparing of the genu of the corpus callosum during WBRT for brain metastases (114). Another study underway at the University of California San Diego is investigating sparing of white matter tracts during SRS for brain metastases (115).

In recent years, brain metastasis velocity, which describes the recurrence rate of new brain metastases following treatment with SRS had become a validated prognostic metric (116). NRG BN009 is a phase 3 trial comparing salvage SRS to SRS with hippocampal avoidance WBRT with the addition of memantine in patients with a first or second distant relapse following upfront SRS and a brain metastasis velocity of 4 or higher (32).

## Conclusion

Cognitive decline is a common clinical manifestation observed in patients who undergo WBRT for brain metastases. Strategies, such as SRS and the addition of memantine and/or hippocampal avoidance to WBRT are excellent treatment options to mitigate this risk. Studies are underway that will allow for further application of these treatments, as well as defining the role that other brain regions play in the pathogenesis of cognitive decline following cranial irradiation.

## Author Contributions

Conceptualization: EL, DT. Supervision: DT. Writing – original draft: EL, BJ, DR. Writing – review and editing: All authors. All authors contributed to the article and approved the submitted version.

## Conflict of Interest

PB reports contribution to UpToDate outside of the submitted work. JW is on the advisory board of Bayer, he serves as a consultant to Angiochem, Bayer, Juno, Novocure, Vanquish Oncology, and GT Medical technologies. JP reports research funding and honoraria from Varian and research funding from Genentech, NIH, and Kroger; he serves on the advisory board of Novocure. IM serves as a consultant to BrainLab and Integra; DT reports institutional support from Novocure Ltd and consulting for Boston Scientific Corporation outside to the submitted work.

The remaining authors declare that the research was conducted in the absence of any commercial or financial relationships that could be construed as a potential conflict of interest.

## Publisher’s Note

All claims expressed in this article are solely those of the authors and do not necessarily represent those of their affiliated organizations, or those of the publisher, the editors and the reviewers. Any product that may be evaluated in this article, or claim that may be made by its manufacturer, is not guaranteed or endorsed by the publisher.
